# NUCB2/Nesfatin-1 Reduces Obesogenic Diet Induced Inflammation in Mice Subcutaneous White Adipose Tissue

**DOI:** 10.3390/nu14071409

**Published:** 2022-03-28

**Authors:** Seley Gharanei, Manjunath Ramanjaneya, Aaran Hitesh Patel, Vanlata Patel, Kiran Shabir, Callum Auld, Emmanouil Karteris, Ioannis Kyrou, Harpal Singh Randeva

**Affiliations:** 1 Warwickshire Institute for the Study of Diabetes, Endocrinology and Metabolism (WISDEM), University Hospitals Coventry and Warwickshire NHS Trust, Coventry CV2 2DX, UK; s.gharanei@warwick.ac.uk (S.G.); aaran.patel@warwick.ac.uk (A.H.P.); kiran.shabir@warwick.ac.uk (K.S.); 2 Warwick Medical School, University of Warwick, Coventry CV4 7AL, UK; m.ramanjaneya@warwick.ac.uk (M.R.); v.h.patel@warwick.ac.uk (V.P.); 3 Qatar Metabolic Institute, Hamad Medical Corporation, Doha P.O. Box 3050, Qatar; 4 Translational Research Institute, Hamad Medical Corporation, Doha P.O. Box 3050, Qatar; 5 Altnagelvin Hospital, Derry BT47 6SB, UK; calmacauld@gmail.com; 6 College of Health, Medicine and Life Sciences, Brunel University London, Uxbridge UB8 3PH, UK; emmanouil.karteris@brunel.ac.uk; 7 Centre for Sport, Exercise and Life Sciences, Research Institute for Health & Wellbeing, Coventry University, Coventry CV1 5FB, UK; 8 Laboratory of Dietetics and Quality of Life, Department of Food Science and Human Nutrition, School of Food and Nutritional Sciences, Agricultural University of Athens, 11855 Athens, Greece; 9 Aston Medical School, College of Health and Life Sciences, Aston University, Birmingham B4 7ET, UK

**Keywords:** adipose tissue, inflammation, nesfatin-1/NUCB2, high-fat high-sugar diet, obesogenic diet, obesity, NF-κB, HMGB1

## Abstract

Background: Excess adipose tissue accumulation and obesity are characterised by chronic, low-grade, systemic inflammation. Nestfatin-1 is a neuropeptide derived from the precursor protein nucleobindin-2 (NUCB2), which was initially reported to exert anorexigenic effects. The present study aimed to investigate the effects of an obesogenic diet (OD; high-fat, high-sugar) in NUCB2 knockout (KO) mice and of nesfatin-1 treatment in LPS-stimulated 3T3-L1 preadipocytes. Methods: Subcutaneous white adipose tissue (Sc-WAT) samples from wild type (WT) and NUCB2 KO mice that were fed a normal diet (ND), or the OD for 12 weeks were used for RNA and protein extraction, as well as immunohistochemistry. 3T3-L1 cells were treated with 100 nM nesfatin-1 during differentiation and stimulated with 1 µg/mL LPS for measuring the expression and secretion of pro-inflammatory mediators by qPCR, western blotting, immunofluorescence, Bioplex, and ELISA. Results: Following the OD, the mRNA, protein and cellular expression of pro-inflammatory mediators (Tnfα, Il-6, Il-1β, Adgre1, Mcp1, TLR4, Hmbgb1 and NF-kB) significantly increased in the ScWAT of NUCB2 KO mice compared to ND controls. Adiponectin and Nrf2 expression significantly decreased in the ScWAT of OD-fed NUCB2 KO, without changes in the OD-fed WT mice. Furthermore, nesfatin-1 treatment in LPS-stimulated 3T3-L1 cells significantly reduced the expression and secretion of pro-inflammatory cytokines (Tnfα, Il-6, Il-1β, Mcp1) and hmgb1. Conclusion: An obesogenic diet can induce significant inflammation in the ScWAT of NUCB2 KO mice, involving the HMGB1, NRF2 and NF-kB pathways, while nesfatin-1 reduces the pro-inflammatory response in LPS-stimulated 3T3-L1 cells. These findings provide a novel insight into the metabolic regulation of inflammation in WAT.

## 1. Introduction

Adipose tissue consists of adipocytes, macrophages, fibroblasts, mesenchymal cells, pre-adipocytes, and endothelial cells, and is involved in the regulation of a wide range of body functions ranging from metabolic homeostasis and appetite to inflammation [1,2]. Obesity is characterized by an accumulation of excess adipose tissue and, in parallel, the progressive development of a chronic state of low-grade, systemic inflammation [3,4]. The sequelae of this inflammation include development of insulin resistance, type 2 diabetes mellitus (T2DM), dyslipidaemia, and cardiovascular disease (CVD). Obesity-related inflammation is associated with dysfunctional adipocytes and the infiltration of adipose tissue depots by an increasing number of immune cells, mostly macrophages, which secrete an array of pro-inflammatory adipokines and cytokines [5].

Nesfatin-1 is a satiety-inducing adipokine with a role in energy balance regulation [6]. The NUCB2 gene encodes a 396 amino acid (aa) long precursor peptide, as well as a 24 amino acid long signal peptide. NUCB2 is cleaved into three fragments by prohormone/proprotein convertase (PC) 1/3 and PC2, to produce nesfatin-1 (aa 1–82), nesfatin-2 (aa 85–163) and nesfatin-3 (aa 166–396) [6,7]. NUCB2/nesfatin-1 is abundantly expressed in several regions of the hypothalamus that control food intake [8]. Indeed, it has been shown that NUCB2/nesfatin-1 reduces food intake in rodents when administrated either centrally or peripherally [6,9]. Of note, NUCB2/nesfatin-1 is also widely distributed in various other tissues [10], and we have previously shown that the white adipose tissue (WAT)—specifically the subcutaneous (Sc) white adipose tissue depot—is a source of peripheral nesfatin-1 [11].

Anti-inflammatory actions of nesfatin-1 have also been demonstrated in several tissues, including in human and murine models of acute lung inflammation [12,13]. Indeed, Hui et al. (2021) showed that NUCB2/nesfatin-1 reduces inflammation in a mouse model of acute lung injury induced by lipopolysaccharide (LPS, also known as endotoxin), through inhibiting neutrophil accumulation and inflammatory cytokine expression [12]. Similarly, Wang et al. (2020) reported that nesfatin-1 reduces murine endotoxin-induced lung inflammation via downregulation of HMGB1, resulting in reduced activity of the mitogen-activated protein kinase (p38MAPK) and nuclear factor kappa B (NF-κB) pathways [13]. Previously, a positive correlation between NUCB2/nesfatin-1 and systemic inflammation was shown in a human study on chronic obstructive pulmonary disease (COPD) by Leivo-Korela et al. [14]. Furthermore, a study with NUCB2 knockout (KO) mice by Ravussin et al. (2018) showed that loss of *Nucb2* aggravated metabolic inflammation in adipose tissue macrophages upon high fat diet (HFD) feeding in an NF-κB-dependent manner, without inducing a classical M1 or alternative M2-like macrophage polarization [15]. This study also showed that loss of NUCB2 in myeloid cells, but not in adipocytes, mediated insulin resistance noted in response to HFD. However, contradictory data exists on the role of nesfatin-1 in chondrocytes. As such, Jiang et al. (2020) reported anti-inflammatory and anti-apoptotic effect of nesfatin-1 in rodent chondrocytes; demonstrating that nesfatin-1 suppressed the IL-1β-induced activation of NF-κB, the MAPK, and the Bax/Bcl-2 signalling pathway in chondrocytes [16]. Scotece et al. (2014), to the contrary, reported pro-inflammatory properties of NUCB2/nesfatin-1 in human and murine chondrocytes [17].

In obesity-induced inflammation, there is an apparent interplay between HMGB1, NRF2 and NF-κB signalling pathways [18,19]. HMGB1 is a highly conserved protein that is ubiquitously expressed in the nucleus of many cell types, including macrophages, neuronal cells, and adipocytes [20]. In response to inflammation or damage, HMGB1 translocates to the cytoplasm and binds to Toll-like receptor 4 (TLR4) to mediate HMGB1-dependent cytokine release involving NF-κB. Activated NF-κB acts as a transcription factor for genes regulating the production of inflammatory cytokines and mediators [21,22,23]. Inhibition of HMGB1 activates the NRF2 pathway that leads to the inhibition of NF-κB inflammatory activity; thus NRF2 activation is accompanied by the downregulation of HMGB1 [19].

Although we have previously reported anti-inflammatory effects of nesfatin-1 in adipose tissue [11], there is still a paucity of data on the exact role of nesfatin-1 in WAT inflammation. Therefore, the present study aimed to investigate the potential role of nesfatin-1 in OD-induced adipose tissue inflammation in wild-type and *Nucb2* knockout mice. Specific focus was placed on the activation of the NRF2 signalling pathway, which is reported to be associated with the inhibition of the NF-κB signalling pathway via HMGB1 inhibition [24]. Thus, it was hypothesized that the inflammation due to adipose tissue expansion in obesity could be exacerbated by *Nucb2* knockdown and lead to upregulation of HMGB1. This would subsequently result in NF-κB pathway activation and the blocking of NRF2 activity. We further expanded our study to investigate potential anti-inflammatory effects of nesfatin-1 in vitro in LPS-stimulated 3T3-L1 cells.

## 2. Materials and Methods

### 2.1. Animals

NUCB2 KO mice were purchased from Jackson laboratories (USA) and adult male C57BL/6J wild-type mice were obtained from Harlan UK, Ltd., Derby, UK. or by inbreeding in-house. All experiments were carried out in accordance with the United Kingdom Home Office Guide on the Operation of Animal (Scientific Procedures) Act of 1986, UK. All mice were housed individually or in groups of two/three. The mice were maintained under pathogen-free conditions with controlled temperature and humidity, and a 12-h light (0700–1900 h), 12-h dark (1900–0700 h) cycle. In addition, the mice were provided with free access to standard mice diet and water. For the 12-week experiments of this study, both the standard normal diet (ND) and the OD were obtained from RMI, Dietex International Ltd., Essex, UK. The ND consisted of 70% carbohydrate, 20% protein, and 10% fat, with an energy density of 3.85 kcal/g, whilst the OD consisted of 40% fat (31% lard and 9% corn oil), 45% carbohydrate (15.2% corn starch, 5% cellulose, and 20% sucrose), and 15% protein (casein), with an energy density of 4.71 kcal/g.

Mice were deprived of food for 12 h before sacrifice by cervical dislocation between 9.00 and 10.30 a.m. Samples of WAT were dissected, and other tissue/organ samples were collected. Only male mice (*n* = 10 in each group) were used in this study. All tissues were immediately dropped in liquid nitrogen and then stored at −80 °C until further analyses.

### 2.2. RNA Extraction and RT-qPCR

Total RNA was extracted from frozen tissue samples using the Fatty tissue RNA Isolation kit (36240, Norgen Biotek Corp., Thorold, ON, Canada), and from cells using the Qiazol lysis reagent (Qiagen, Hilden, Germany). Equal amounts of total RNA (1 µg) were reverse transcribed using the high-capacity Reverse Transcription Kit (Life technology, Carlsbad, CA, USA), and the resulting cDNA was used as template in RT-qPCR analysis. Detection of gene expression was performed using the Taqman gene expression assays with Fast Taqman Advanced mastermix (4444557, Fisher Scientific, Hampton, NH, USA) or SYBR Green technology (SYBR Green Jump start taq ready mix, S4438, Merck). Details of the Taqman gene expression assays and qPCR primers are presented in Table 1. The ABI 7500 Real-Time PCR or Quant studio PCR System (Applied Biosystems, Waltham, MA, USA) were utilized for these experiments. The expression levels of the analysed samples were calculated using the ∆Ct method, and the variances of input cDNA were normalised against the levels of the beta-actin or L19 housekeeping genes. All measurements were performed in triplicate.

### 2.3. Western Blotting

Protein was extracted from frozen tissue samples using RIPA buffer and a hand homogeniser. Protein content was determined by BCA Protein Assay (#23225; Fisher Scientific, Loughborough, UK), following the manufacturer’s instructions. Samples (25 μg) were prepared in 20% (v:v) Lane Marker Reducing Sample Buffer 5 × (Fisher Scientific), and heated at 95 °C for 5 min. Lysates were loaded into 10% mini polyacrylamide gels (Biorad, Hercules, CA, USA) and separated by standard SDS-PAGE electrophoresis. Proteins were transferred onto a 0.45 micron PVDF Membrane at 300 mA for 75 min (GE Healthcare, Amersham, UK) and blocked in 2% BSA (w:v) in TBS-T (50 mM Tris, 150 mM NaCl, 0.5% (v:v) Tween-20, pH 7.4) for 1 h. Membranes were incubated with primary antibodies overnight at 4 °C (Table 2). Blots were washed clear of unbound antibody in TBS-T before addition of anti-mouse or anti-rabbit-HRP conjugated secondary antibody (1:5000 in TBS-T; Cell Signalling Technologies, Danvers, MA, USA) for 1 h at RT. Un-bound antibody was washed in TBS-T and immune-reactive bands visualized with ECL prime reagent (GE Healthcare, Amersham, UK) and standard auto-radiography techniques. The density of individual bands was determined using the ImageJ gel analysis software.

### 2.4. Cell Culture

3T3-L1 cells [11] were grown in DMEM media supplemented with 10% foetal bovine serum (FBS; Sigma, Welwyn Garden City, UK) and 1% P/S (penicillin/streptomycin, Thermo Fisher Scientific, Waltham, MA, USA). Upon 80% confluency, cells were differentiated in growth media supplemented with 1 µg/mL insulin, 0.25 µM Dexamethasone, 0.5 mM IBMX for 4 days, and were further maintained in growth media supplemented with 1 µg/mL insulin for 6 days. Cells were treated with nesfatin-1 (100 nM) [13,16] during the differentiation and were stimulated with LPS (1 µg/mL) [13,25] for 5 hr and 24 hr and for 1 h for the detection of phosphorylated proteins.

### 2.5. Immunofluorescence

Mouse ScWAT biopsies were fixed overnight in 10% neutral buffered formalin at 4 °C and wax embedded in Surgipath Formula “R” paraffin using the Shandon Excelsior ES Tissue processor (Thermo Fisher Scientific). Tissues were sliced into 3 µM sections on a microtome and adhered to super adhesive slides by overnight incubation at 60 °C. Slides were rehydrated and underwent deparaffinization by antigen retrieval (sodium citrate buffer; 10 mM sodium citrate, 0.05% Tween20, pH 6). Sections were treated with 3% H_2_O_2_ for 10 min and blocked with 2% BSA in TBST for 30 min. Sections were subsequently incubated in primary antibody (Table 1) at 4 °C overnight, followed by incubation with Alexa Fluor 488 anti-rabbit or 555 anti-mouse secondary antibody for 1 h at RT (1:1000; Fisher Scientific). Slides were mounted using super gold anti-fading mounting media with DAPI (Vector Laboratories, Burlingame, CA, USA) to visualize the nuclei. Images were visualised and captured on EVOS AUTO microscope (Thermo Fisher Scientific).

3T3-L1 cells were grown on 8 chamber slides, fixed with 4% paraformaldehyde (Thermo Fisher Scientific) for 10 min, permeabilised in 0.2% Triton-X 100 (Sigma, Welwyn Garden City, UK) and blocked with 2% BSA in TBST for 30 min.

### 2.6. Bioplex-200 and ELISA

3T3-L1 cell supernatants were collected, centrifuged at 1500 rpm for 10 min at 4 °C and stored in −80 °C until further use. These samples were used to measure the secretory levels of MCP-1 and IL6 by Bioplex-200 using a mouse cytokine assay (Biorad Technologies, UK), whilst the secretory levels of HMGB1 were measured using a mouse HMGB1 ELISA kit (NBP2-62767, Biotechne, Abingdon, UK) according to the manufacturer’s instructions.

### 2.7. Statistical Analysis

Data were analysed with the statistical package GraphPad Prism 9 (GraphPad Software Incorporated, La Jolla, CA, USA). Two-way ANOVA was used for mice data and One-way ANOVA with post hoc Tukey’s test were used for 3T3-L1 data and statistical significance was set at *p*-value < 0.05.

## 3. Results

### 3.1. Adipose Tissue of Nucb2 KO Mice on OD Show Aggravated Inflammation

**Figure 1:**
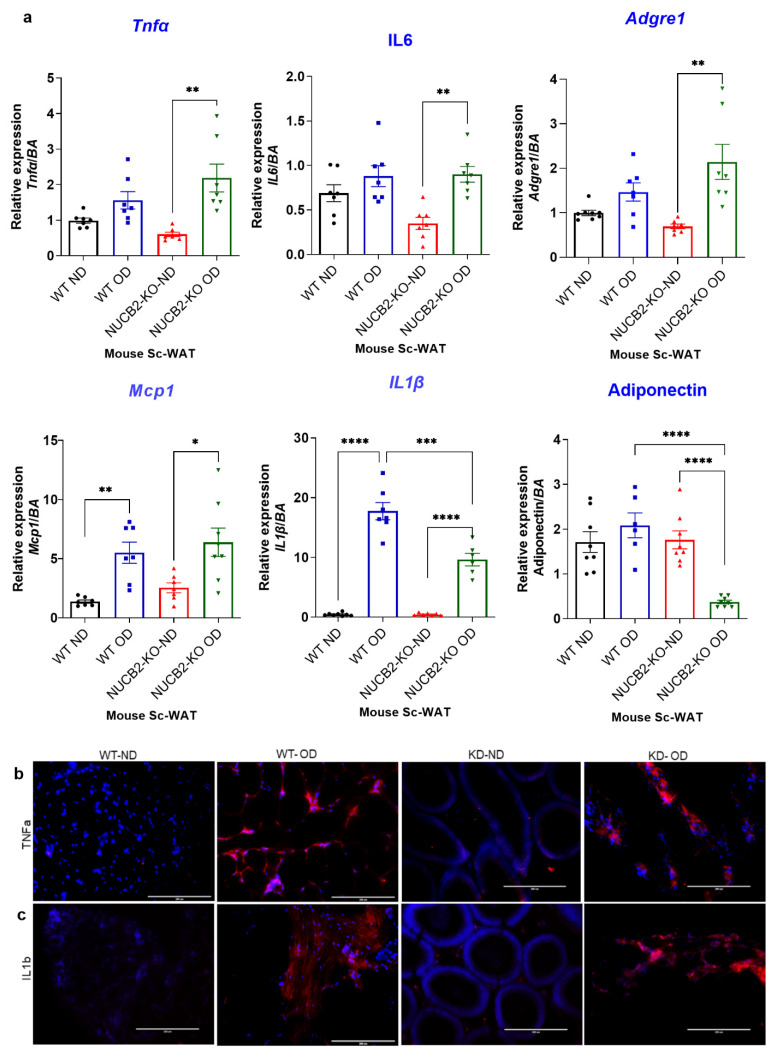
A 12-week obesogenic diet (OD) induced a pro-inflammatory response in the subcutaneous white adipose tissue (ScWAT) of Nucb2 knockout (KO) and wild type (WT) mice. (**a**) mRNA levels of Tnfa, Il6, Il1b, Adgre1, Mcp1, and adiponectin were determined in the ScWAT of Nucb2 KO and WT mice on normal diet (ND) and following a 12-week OD. Data are presented as fold-change (mean ± SEM) in transcript, and six to seven mice were used in each group. Two-way ANOVA; any noted statistically significant difference for the pairwise comparisons (WT ND vs. WT OD, KO ND vs. KO OD, WT ND vs. KO ND and WT OD vs. KO OD) is presented as: * (*p* < 0.05); ** (*p* < 0.01); or *** (*p* < 0.001); ****(<0.0001). Immunofluorescence staining of (**b**) TNFA and (**c**) IL1B (red) in ScWAT from WT and Nucb2 KO mice on ND and OD. DAPI (blue) was utilized to stain the nuclei. Photos were taken at 20× magnification on EVOS fluorescence microscope, scale bar 200 µm.

Adipose tissue is known to secrete pro-inflammatory molecules through activation of TLR4 [26]. To determine the anti-inflamatory effects of Nucb2/nesfatin-1 on adipose tissue, we analysed the expression and secretion of pro-inflammatory mediators in Nucb2 KO and WT mice following 12 weeks of an OD. The weight of the mice during this feeding period is presented in Supplementary Figure S1. Gene expression of pro-inflammatory cytokines Tnfa, Il6, Il1b, Mcp1 was measured in ScWAT of WT and Nucb2 KO mice (ND and OD). Tnfa and Il-6 levels significantly increased in OD-fed Nucb2 KO mice compared to those on ND (*p* = 0.0018 and *p* = 0.0061, recpectively; Figure 1a). No significant changes were seen in Tnfa and Il-6 levels for the WT mice fed the 12-week OD compared to those on ND. However, the expression levels of Il1b and Mcp1 were significantly elevated in the ScWAT of both WT (*p* < 0.0001 and *p* = 0.0059, respectively; Figure 1a) and Nucb2 KO mice (*p* < 0.0001, and *p* = 0.0173, respectively; Figure 1a) with the OD feeding in comparison to their counterparts on ND. We also measured the expression of the anti-inflammatory adipokine adiponectin, which showed a significant attenuation in Nucb2 KO on OD (*p* < 0.0001, Figure 1a), but no significant changes in the WT on OD. Furthermore, the expression of Adgre1 (F4/80) was significantly elevated in Nucb2 KO on OD compared to ND (*p* = 0.0029, Figure 1a) with no significant changes in the WT between HFD and ND.

Inflammatory cytokines protein expression was also investigated by immunofluorescence, where the expression of TNFα and IL-1β appeared elevated in OD models (Figure 1b,c). Overall, these data indicate that loss of Nucb2/nesfatin-1 in the presence of a OD is associated with increased inflammation in ScWAT.

### 3.2. Hmgb1 and NF-κB Upregulation with OD Results in Attenuation of Nrf2 Expression in Nucb2 KO ScWAT

**Figure 2:**
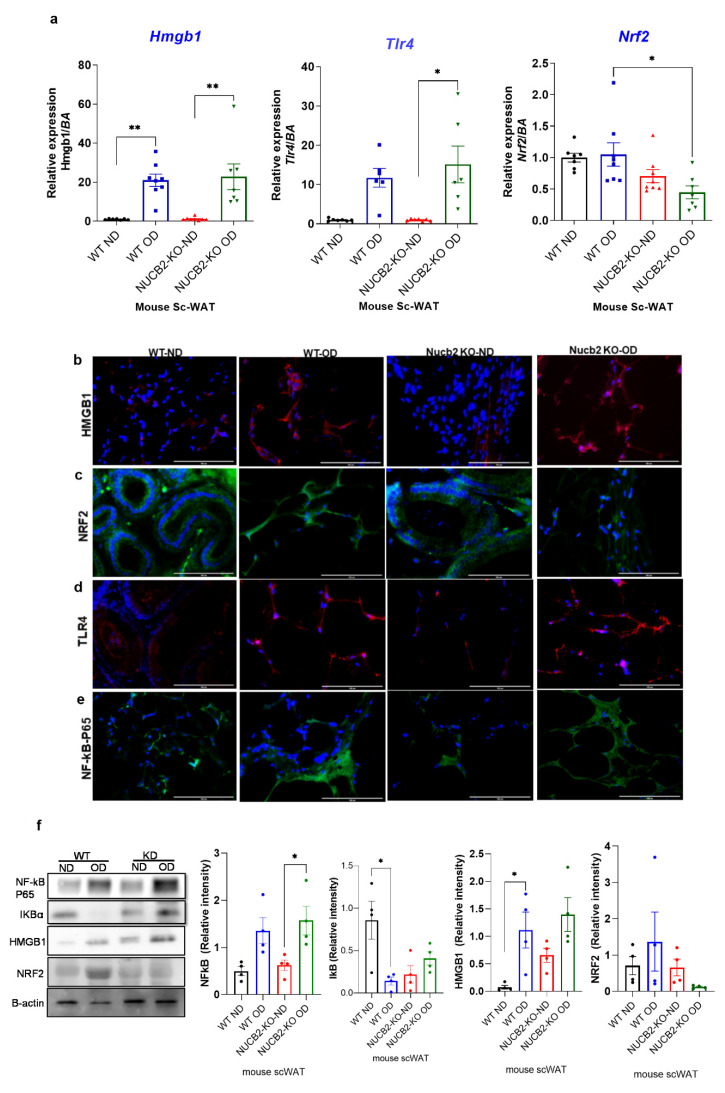
Hmgb1 and NF-kB upregulation following a 12-week obesogenic diet (OD) results in NRF2 reduced activity in the subcutaneous white adipose tissue (ScWAT) of Nucb2 knockout (KO) mice. (**a**) Hmgb1, Tlr4 and Nrf2 mRNA levels were determined in the ScWAT of Nucb2 KO and wild type (WT) mice on normal diet (ND) and following a 12-week OD. Data are presented as fold-change (mean ± SEM) in transcript, and six to seven mice were used in each group. Two-way ANOVA; any noted statistically significant difference for the pairwise comparisons (WT ND vs. WT OD, KO ND vs. KO OD, WT ND vs. KO ND and WT OD vs. KO OD) is presented as: * (*p* < 0.05); ** (*p* < 0.01) Immunofluorescence staining of (**b**) HMGB1 (red), (**c**) NRF2 (green), (**d**) TLR4 (red) and (**e**) NF-kB p65 (green) in ScWAT from WT and Nucb2 KO mice on ND and OD. DAPI (blue) was utilized to stain the nuclei. Photos were taken at 40× magnification on EVOS fluorescence microscope, scale bar 100 µm. (**f**) NF-kB p65, IkB, HMGB1 and NRF2 protein levels were determined by Western blot analysis, normalized to beta actin levels, and relative intensity was determined by densitometry. N of 4 animals were used per group, Two-way ANOVA; any noted statistically significant difference for the pairwise comparisons (WT ND vs. WT OD, KO ND vs. KO OD, WT ND vs. KO ND and WT OD vs. KO OD) is presented as: * (*p* < 0.05).

The anti-inflammatory effects of nesfatin-1 have been attributed to the downregulation of HMGB1 in endotoxin induced lung inflammation [13]. Similarly, HMGB1 inhibition is associated with activation of the NRF2 signaling pathway, which leads to the inhibition of the NF-κB signalling pathway [24]. Thus, in the present study we also investigated the gene expression of Hmgb1, Nrf2 and Tlr4 in ScWAT. Our findings show that the expression of Hmgb1 was significantly increased with the 12 weeks OD feeding in both the WT and Nucb2 KO mice in comparison to the ND-fed mice (Hmgb1: *p* = 0.0024, and *p* = 0.0011 respectively Figure 2a). Tlr4 expression was significantly increased in the Nucb2 KO on OD in comparison to ND (*p* = 0.01), and with no significant changes in the OD-fed WT mice compared to those on ND (Figure 2a). However, Nrf2 expression was significantly decreased only in the Nucb2 KO on OD in comparison to WT on OD (*p* = 0.04, Figure 2a), with no significant changes observed in the OD-fed mice compared to those on ND in both groups.

Figure 2b–e presents the immunofluorescence staining of NF-κB, HMGB1, NRF2, and TLR4 in the ScWAT of Nucb2 KO and WT mice on ND and HFD. HMGB1 expression appeared weak in ND and an intense cytoplasmic expression was observed in the ScWAT of both the OD-fed WT and Nucb2 KO (Figure 2b). NRF2 expression was present in the ScWAT of WT mice on ND, and seems to increase with the OD. Moreover, NRF2 expression was also evident in the ScWAT of Nucb2 KO mice on ND, but was clearly weaker with the OD (Figure 2c). TLR4 expression also appeared weak in the ND ScWAT samples, and intense in the OD ScWAT samples, with more pronounced expression in the OD-fed Nucb2 KO mice (Figure 2d). The expression of NF-κB seemed weak in mice fed a ND and increased with the OD. Notably, NF-κB expression in the ScWAT tissue of WT mice fed OD appears predominantly cytoplasmic, whereas NF-κB seems to be present in and around the nucleus in Nucb2 KO mice on OD (Figure 2e). Collectively, these results indicate that in lack of NUCB2/nesfatin-1 under obesogenic conditions activates NF-kB resulting in its translocation to the nucleus. Similarly, these results suggest that increased cytoplasmic HMGB1 is possibly released extracellularly, where it binds to TLR4 and activates it [27]. Likewise, these results suggest that reduced expression of NRF2 in the ScWAT of the OD-fed Nucb2 KO mice indicate reduced activity of the NRF2 pathway.

On protein level, the expression of NF-κB p65 and its inhibitor IkB, as well as that of HMGB1 and NRF2, were measured by western blotting. Figure 2f presents an upregulation in NF-κB p65 protein expression in ScWAT of WT and Nucb2 KO mice fed OD (*p* = 0.03). The expression of IkB was significantly decreased in the ScWAT samples from OD-fed WT mice compared to ND counterparts (*p* = 0.02), with no significant change between the ScWAT samples from ND and OD-fed Nucb2 KO mice. These results indicate that lack of NUCB2 contibutes to exacerbated OD-induced inflammation in ScWAT through activation of the NF-κB pathway. Similarly, HMGB1 protein expression appeared to be weak in the ScWAT of ND-fed WT mice, and increased with OD (*p* = 0.02), whereas, HMGB1 is exressed in the ScWAT of ND-fed Nucb2 KO mice and was upregulated with OD, but not significantly (Figure 2f). NRF2 protein expression showed no significant change in the ScWAT of OD-fed WT mice compared to ND, whereas it was substantially reduced in the ScWAT of OD-fed Nucb2 KO mice compared to ND, but not significantly (Figure 2f). As such, these findings suggest that under OD-induced inflammation, lack of Nucb2 upregulates HMGB1 via the activation of TLR4, resulting in attenuation of NRF2.

### 3.3. Nesfatin-1 Protects against Endotoxin Induced Inflammation

Finally, we investigated the anti-inflammatory effects of nesfatin-1 in a mouse white adipocyte cell line (3T3-L1). These cells were treated with nesfatin-1 (100 nM) during differentiation and were stimulated with LPS (1 µg/ML). The LPS time course was optimized for mRNA and protein level (Supplement Figure S2), with optimum upregulation of cytokine gene expression being observed after 4 h of treatment and optimum phosphorylation occurring after 1 h.

**Figure 3:**
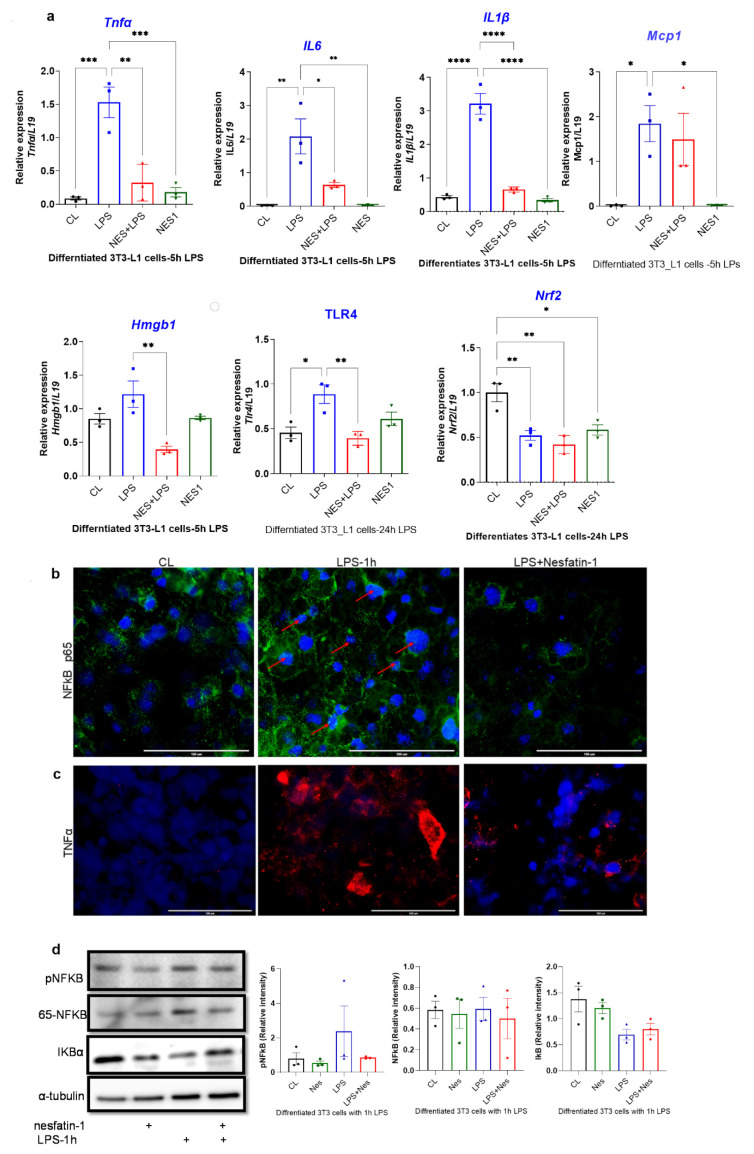

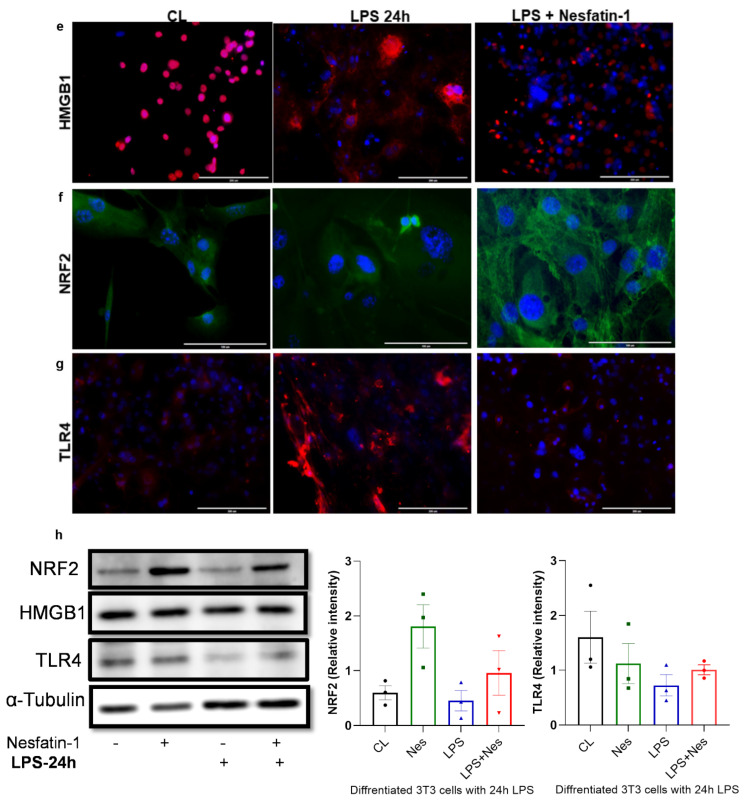
Nesfatin-1 protects against lipopolysaccharide (LPS, also known as endotoxin) induced inflammation. (**a**) mRNA levels of Tnfa, Il6, Il1b, Mcp1, and Hmgb1 were determined in differentiated 3T3-L1 cells with and without nesfatin-1 treatment, as well as with and without 5 h LPS treatment and the combination of the two treatments. The mRNA expression of NRF2 and TLR4 were measured following a 24 h LPS treatment. Data are presented as fold-change (mean ± SEM) in transcript. The experiments were repeated in three independent cultures. One-way ANOVA; * *p* < 0.05; ** *p* < 0.01; *** *p* < 0.001; p****<0.0001. Immunofluorescence staining of (**b**) TNFa (red) and (**c**) NF-kB (green) in differentiated 3T3-L1 cells with and without nesfatin-1 treatment, as well as with and without 1 h LPS treatment and the combination of the two treatments. DAPI (blue) was utilized to stain the nuclei, arrows indicate presence of NF-kB in the nuclei. Photos were taken at 40× magnification on EVOS fluorescence microscope, scale bar 100 µm. (**d**) p-NF-KB, total NF-kB, and IKB protein levels were determined by western blot analysis in differentiated 3T3-L1 cells with and without nesfatin-1 treatment, as well as with and without 1 h LPS treatment and the combination of the two treatments, normalized to α-tubulin levels, and relative intensity was determined by densitometry. The experiments were repeated in three independent cultures, One-way ANOVA; Immunofluorescence staining of (**e**) HMGB1 (red), (**f**) TLR4 (red) and (**g**) NRF2 (green) in differentiated 3T3-L1 cells with and without nesfatin-1 treatment, as well as with and without 1 h LPS treatment and the combination of the two treatments. DAPI (blue) was utilized to stain the nuclei. Photos were taken at 20× or 40× magnification on EVOS fluorescence microscope, scale bar 100 or 200 µm. (**h**) HMGB1, NRF2 and TLR4 protein levels were measured in these samples after 24 h of LPS treatment. Expression was normalized to α-tubulin levels, and relative intensity was determined by densitometry. The experiments were repeated in three independent cultures.

The expression of inflammatory cytokines in nesfatin-1 treated 3T3-L1 cells with and without 5 h of LPS stimulation, as well as the expression of *Hmgb1*, *Tlr4* and *Nrf2* following a 24 h LPS treatment was assessed. The expression of *Tnfa*, *Il6*, *Mcp-1* and *Il1b* were significantly elevated with LPS stimulation in comparison to untreated controls (*p* = 0.0005, *p* = 0.0025, *p* = 0.026, *p* < 0.0004, respectively; Figure 3a). However, pre-treatment with nesfatin-1 significantly downregulated the LPS-induced expression of *Tnfa*, *Il6* and *Il1b* (*p* = 0.0016, *p* = 0.019, *p* < 0.0001, respectively), but not of *Mcp-1* (Figure 3a). The expression of *Hmgb1* was significantly increased after 5 h of LPS treatment in comparison to untreated cells (*p* < 0.0001, Figure 3a). Pre-treatment with nesfatin-1, significantly reduced the LPS-induced *Hmgb1* expression (*p* = 0.003, Figure 3a). The mRNA expression of Nrf2 and Tlr4 was also measured after 24 h LPS treatment. Significant downregulation of *Nrf2* levels were observed with nesfatin-1 treatment alone, LPS alone or in combination compared to control cells (*p* < 0.05, Figure 3a). Tlr4 mRNA levels were significantly increased after the 24 h LPS treatment in comparison to untreated cells (*p* = 0.015 Figure 3a). Pre-treatment with nesfatin-1, significantly reduced the LPS-induced *Tlr4* expression (*p* = 0.007, Figure 3a).

Differentiated and nesfatin-1 treated 3T3-L1 cells were either treated for 1 h with LPS to measure phosphorylated proteins or for 24 h to measure nuclear proteins by immunofluorescence and immunoblotting. Following 1 h LPS treatment, NF-κB expression appeared to be elevated in LPS treated cells, with the expression being evident in the nuclei. Pre-treatment with nesfatin-1 seemed to have reduced NF-κB expression with no expression in the nuclei (Figure 3b). Similarly, TNFα staining seemed visibly increased in cells treated with LPS for 1 h. However, the presence of nesfatin-1, weakened the staining in LPS-stimulated cells (Figure 3c). Immunoblotting data shows that the expression of phosphor-NF-κB protein was increased in LPS-treated samples, and reduced with nesfatin-1 treatment, whilst there was no significant change in samples treated with nesfatin-1 plus LPS. The expression of total NF-κB protein was increased with LPS treatment, while it was reduced in cells pre-treated with nesfatin-1. In addition, the expression of the NF-κB inhibitor Iκb is reduced with LPS stimulation and increased with nesfatin-1 treatment (Figure 3d). Changes on protein level were very subtle, but evident in triplicate samples (*n* = 3).

Immunofluorescence staining of HMGB1, NRF2 and TLR4 following a 24 h LPS treatment is presented in Figure 3e–g. Based on this staining, HMGB1 appears highly expressed in differentiated 3T3-L1 cells exclusively in the nucleus. Moreover, the 24 h LPS treatment resulted in extensive cytoplasmic expression. However, in cells pre-treated with nesfatin-1, this LPS treatment had a limited effect, with cells maintaining their nuclear HMGB1 expression (Figure 3e). Furthermore, NRF2 seems to be expressed in differentiated 3T3-L1 cells, with a 24 h LPS treatment resulting in reduced NRF2 expression. In cells pre-treated with nesfatin-1, the NRF2 expression seems to be intensified in the cytoplasm, with the applied LPS treatment having no significant effect on this (Figure 3f). Moreover, TLR4 expression seems weak in untreated cells, highly increased following the 24 h LPS treatment, and considerably reduced in cells pre-treated with nesfatin-1 (Figure 3h).

The protein expression of NRF2, HMGB1, and TLR4 was also measured by western blot following a 24 h LPS treatment. Nesfatin-1 treatment resulted in upregulation of NRF2 expression, with and without LPS treatment, whereas HMGB1 protein expression did not changed throughout these treatments (Figure 3h). As HMGB1 is highly expressed in differentiated 3T3-L1 cells [27,28], LPS treatment would translocate the protein to the cytoplasm [28] as shown in Figure 3e, whilst the total protein level is not changed. TLR4 expression measured by western blot provided opposing results, showing reduced expression with LPS treatment and increased expression with nesfatin-1 treatment (Figure 3h). These contradictory results may be due to differences in TLR4 surface and intracellular levels.

**Figure 4:**
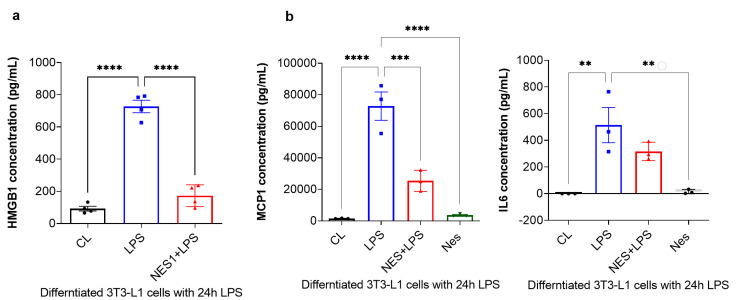
Nesfatin-1 inhibits HMGB1, as well as MCP-1 and IL6 secretion. (**a**) HMGB1 secreted levels were measured by Elisa and the experiments were repeated in four independent cultures. One-way ANOVA; **** *p* < 0.0001. (**b**) MCP-1 and IL6 secreted levels were measured by Bioplex-200 multiplex immunoassays. The experiments were repeated in three independent cultures. One-way ANOVA; ** *p* < 0.01; *** *p* < 0.001, **** *p* < 0.0001.

Finally, the secreted levels of HMGB1 were measured by ELISA, whist MCP-1 and IL6 levels were measured by a multiplex immunoassay using the Bioplex system. As presented in Figure 4a, HMGB1 secretion was significantly increased with 24 h LPS treatment (*p* < 0.0001, Figure 4a). Pre-treatment with nesfatin-1 significantly inhibited HMGB1 secretion with 24 h LPS treatment (*p* < 0.0001, Figure 4a), suggesting that HMGB1 secretion is regulated by nesfatin-1. Furthermore, the secreted levels of MCP-1 and IL6 were significantly increased with the 24 h LPS treatment in comparison to untreated cells (*p* < 0.0001 and *p* = 0.003, respectively; Figure 4b). Pre-treatment with nesfatin-1 significantly reduced MCP-1 secretion in comparison to the LPS treatment (*p* = 0.0006, Figure 4b). IL6 levels were also reduced with nesfatin-1 pre-treatment, but without reaching statistical significance (Figure 4b).

Taken collectively, these results indicate that nefatin-1 protects against LPS-induced inflammation by activating or inducing NRF2 expression and inhibiting HMGB1.

## 4. Discussion

The present study offers novel evidence regarding the anti-inflammatory effects of Nucb2/nesfatin-1 in ScWAT. Indeed, we show significant inflammation in ScWAT of *Nucb2* KO mice following a 12-week OD, with increased expression levels of pro-inflammatory mediators *Tnfa*, *IL6*, *IL1b*, *Mcp1*, *Adgre1*, *Tlr4* and *Hmgb1* and reduced levels of *adiponectin* and *Nrf2*. Our findings also show nuclear translocation of NF-kB and intense cytoplasmic expression of HMGB1 in the ScWAT of OD-fed *Nucb2* KO mice. Furthermore, we showed reduced expression of NRF2 on mRNA, protein, and cellular level in the ScWAT of *Nucb2* KO mice on OD. Our present findings indicate that this increased inflammation is partly mediated through HMGB1 and its downstream signaling pathways; NF-kB and NRF2. Moreover, in 3T3-L1 cells, we demonstrated increased levels of inflammatory mediators, including HMGB1 and NF-kB, in response to LPS (endotoxin) treatment. Notably, pre-treatment with nesfatin-1 significantly reduced this LPS-induced inflammation and was able to significantly inhibit/reduce HMGB1 and MCP-1 secretion in 3T3-L1 cells. In addition, nesfatin-1 reduced the activity of the NF-κB signaling pathway and the antioxidant signaling pathway NRF2, indicating significant anti-inflammatory and antioxidant effects of nesfatin-1 in 3T3-L1 cells. Collectively, our present data suggest that Nucb2/nesfatin-1 acts as an anti-inflammatory adipokine by suppressing HMGB1 and pro-inflammatory cytokine secretion, and promoting NRF2 activation, which leads to inhibition of the inflammatory activity of NF-κB.

Obesity-induced inflammation is associated with insulin resistance and T2DM [4,29]. Activation of the innate immune system has been implicated in WAT inflammation and insulin resistance [30,31]. TLR4 is a cell surface receptor that plays a crucial role in innate immune defense and can engage many pro-inflammatory pathways [32]. HMGB1 acts as an innate pro-inflammatory mediator in WAT of patients with obesity by binding to TLR4 and triggering an inflammatory response [33]. Presence of HMGB1 in inflammatory WAT was attributed to infiltrating macrophages [28]. However, Shimizu et al. (2016) demonstrated that in addition to macrophages, adipocytes also secrete HMGB1 [27]. Indeed, HMGB1 is known to initiate and exacerbate inflammation with increased serum levels reported in obesity [28,34,35]. Our findings are in accord with these previous reports and highlight that Nucb2/nesfatin-1 can inhibit or significantly reduce the pro-inflammatory function of HMGB1. Similarly, Wang and colleagues showed that nesfatin-1 reduces inflammation by regulating the expression of HMGB1, resulting in reduced activity of p38MAPk and NF-kB pathway [13]. Here, we demonstrate that inhibition of HMGB1 is also associated with activation of the NRF2 pathway. The study by Yu et al. (2019) showed that an anti-HMGB1 monoclonal antibody inhibited not only the DNA-binding activity of NF-κB, but also its cytoplasmic-to-nucleus translocation [19]. Glycyrrhizin, a HMGB1 inhibitor, has been shown to activate NRF2, which can also inhibit the inflammatory activity of NF-κB [18]. Of note, NRF2 is known to repress inflammation [36], and a study by Kobayashi et al. (2016) showed that Nrf2 regulates inflammation through transcriptional inhibition of IL6 and IL1b genes [37]. Our data show significant reduction in NRF2 expression in the ScWAT of *Nucb2* KO on OD and in 3T3-L1 cells with LPS treatment, with significant increase with nesfatin-1 treatment, suggesting that nesfatin-1 plays a role in activation of the NRF2 pathway. Interestingly, a recent study demonstrated that the adipokine vaspin was also able to reduce inflammation by downregulation of HMGB1 and activation of NRF2 [24]. In addition, the study by Shimzu et al. (2016) showed that adiponectin protects against HMGB1-induced adipose tissue inflammation by partially suppressing HMGB1 release from adipocytes [27]. Overall, these studies indicate that certain adipokines, including nesfatin-1, vaspin, and adiponectin, can play significant anti-inflammatory roles in metabolically challenging environments.

A limitation of our study is that our functional analyses of nesfatin-1 was confined to the mouse adipose tissue and cell line. Whether nesfatin-1 plays an anti-inflammatory role in human adipose tissue warrants further investigation. Future in vivo studies are needed to investigate further the effect(s) of nesfatin-1 on feeding behavior and inflammation. Moreover, the regulation of nesfatin-1 expression by acute or chronic exposure to different diets (e.g., high-fat, high-carbohydrate and high-protein diets) could potentially affect other organs in different ways [38]. While our study is mainly focused on inflammation induced by an obesogenic diet in the white adipose tissue, future studies should further focus on the effects of obesogenic diets on nesfatin-1 expression and inflammation in other tissues such as the liver, heart and pancreas. In addition, fluorescence image quantification and analysis were outside the scope of our study. Finally, the clinical utility of our findings needs to be further investigated using human models of adiposity (in vivo or ex vivo). Other possible experiments include using human induced pluripotent stem cell (iPSC) derived adipocytes treated with nesfatin-1 and stimulated with LPS in culture. Similar experiments were conducted in human iPSC derived cardiomyocytes [39]. Human primary adipocytes from participants with normal weight and obesity which will be cultured and treated with nesfatin-1 could also provide clues for the anti-inflammatory properties of nesfatin-1. Furthermore, the physiological role of nesfatin-1 in dysmetabolic states, and specifically its role in obesity induced inflammation in vivo, awaits further elucidation.

In conclusion, our present findings suggest that NUCB2/nesfatin-1 partly inhibits HMGB1 expression and secretion, resulting in activation of the NRF2 pathway and subsequent inhibition of NF-κB pro-inflammatory activity. To the best of our knowledge, this is the first study investigating the role of NUCB2/nefatin-1 in white adipose tissue inflammation involving HMGB1, NRF2 and NF-kB pathways. Overall, our present results suggest that nesfatin-1 is an anti-inflammatory adipokine, which may present an additional therapeutic target for protection against the detrimental sequelae of the chronic pro-inflammatory state noted in obesity.

## Figures and Tables

**Table 1:** Mouse Taqman assays/qPCR primers.

	Gene	Assay ID/Primers
1	*TNFa*	Mm99999068_m1
2	*IL6*	Mm00446190_m1
3	*Adgre1*	Mm00802529_m1
4	*Adiponectin*	Mm00456425_m1
5	*Beta-actin*	Mm00607939_s1
6	*HMGB1*	FP:CGGATGCTTCTGTCAACTTCT RP: TGTCAGCCTTTGCCATATCTT
7	*NRF2*	FP: CTGCACTGGAAGGCTACAGA RP: AACCACCCAATGCAGGACTT
8	*MCP-1*	FP: CCAATGAGTAGGCTGGAGAGC RP: ACCCATTCCTTCTTGGGGTC
9	*IL1β*	FP: CACAGCAGCACATCAACAAG RP: GTGCTCATGTCCTCATCCTG
10	*TLR4*	FP: TCTGGGGAGGCACATCTTCT RP: AGGTCCAAGTTGCCGTTTCT
11	*RPL19*	FP: GGAAAAAGAAGGTCTGGTT RP: TGATCTGCTGACGGGAGT
12	*Beta actin*	FP: GCAGGAGTACGATGAGTCCG RP: ACGCAGCTCAGTAACAGTCC

**Table 2:** Details of primary antibodies.

Antibody	Company	Product No	Dilution
p-NF-kB (S536)	Cell Signalling Technologies, Danvers, MA, USA	3031S	WB: 1/500
IkBα	Imagenex, Port Coquitlam, BC, Cannada	IMG-127	WB: 1/1000
NF-kB	Santa Cruz Biotechnology, Dallas, TX, USA	sc-109	WB: 1/1000
NRF2	Santa Cruz Biotechnology	sc-722	WB:1/2000 IHC:1/100
α-Tubulin	GeneTex, Irvine, CA, USA	GTX628802	WB: 1/2000
HMGB1	R&D, Toronto, ON, Cannada	NBP2-25148ss	WB: 1/1000 IF:1/200
TNFα	Abcam, Cambridge, UK	Ab1793	IF: 1/100
IL-1β(3A6)	Cell Signalling Technologies	12242	IF: 1/100
TLR4	Santa Cruz Biotechnology	sc-293072	IF: 1/100
Beta actin	Cell Signalling Technologies	4967	1/1000

## Data Availability

Data are available via the corresponding authors upon reasonable request that does not raise any ethical, privacy, or security concerns.

## Supplementary Materials

The following are available online at https://www.mdpi.com/article/10.3390/nu14071409/s1. Supplementary Figure S1. Average weight gain of the *Nucb2* knockout (KO) and wild type (WT) mice during the experimental period with a 12-week obesogenic diet (OD) and normal diet (ND). OD started at week 8 and continued for 12 weeks. There were eight animals per group. Supplementary Figure S2. Lipopolysaccharide (LPS) optimization time course. (a) *Tnfa*, *Il1b* and *Hmgb1* mRNA levels were determined in differentiated 3T3 L1 cells with a time course of LPS treatment. (b) P-NF-kB levels were determined in differentiated 3T3 L1 cells with a time course of LPS treatment and beta actin was used as a loading control.
