# Supplementary figures and images for: NUCB2/Nesfatin-1 Reduces Obesogenic Diet Induced Inflammation in Mice Subcutaneous White Adipose Tissue
Source: Nutrients. 2022 Mar 28;14(7):1409. doi: 10.3390/nu14071409 (PMC9003550; doi:10.3390/nu14071409)

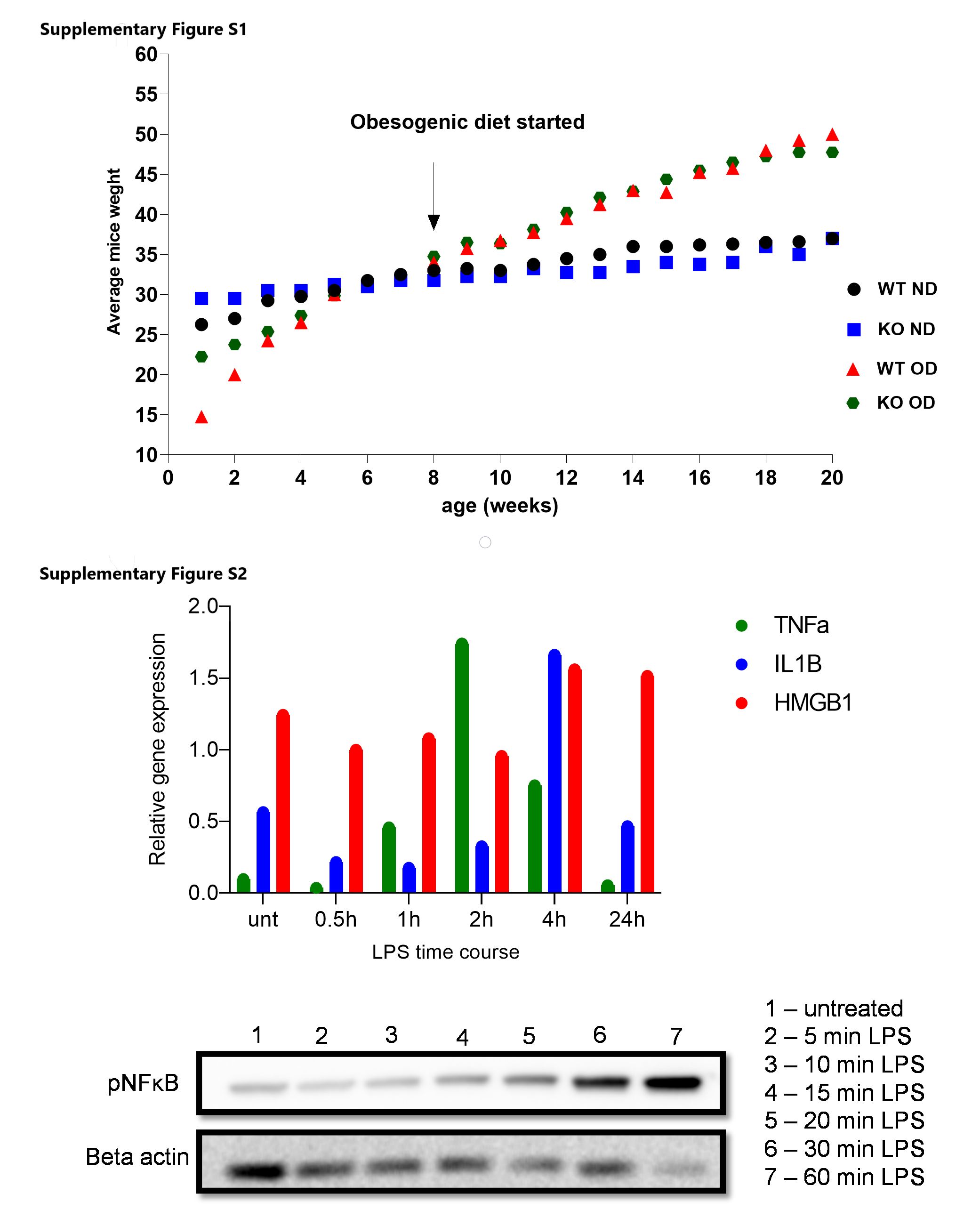

Supplement: Supplementary file 1 [file nutrients-14-01409-s001.zip › nutrients-1605439-supplementary.jpg]
